# Emerging Roles for Mesencephalic Astrocyte-Derived Neurotrophic Factor (MANF) in Pancreatic Beta Cells and Diabetes

**DOI:** 10.3389/fphys.2018.01457

**Published:** 2018-10-16

**Authors:** Tatiana Danilova, Maria Lindahl

**Affiliations:** Institute of Biotechnology, HiLIFE, University of Helsinki, Helsinki, Finland

**Keywords:** MANF, endoplasmic reticulum stress, beta cell, diabetes, knockout mouse, unfolded protein response, regeneration

## Abstract

Mesencephalic astrocyte-derived neurotrophic factor (MANF) was originally identified as a secreted trophic factor for dopamine neurons *in vitro.* It protects and restores damaged cells in rodent models of Parkinson’s disease, brain and heart ischemia, spinocerebellar ataxia and retina *in vivo*. However, its exact mechanism of action is not known. MANF is widely expressed in most human and mouse organs with high levels in secretory tissues. Intracellularly, MANF localizes to the endoplasmic reticulum (ER) and ER stress increases it’s expression in cells and tissues. Furthermore, increased MANF levels has been detected in the sera of young children with newly diagnosed Type 1 (T1D) diabetes and Type 2 (T2D) diabetic patients. ER stress is caused by the accumulation of misfolded and aggregated proteins in the ER. It activates a cellular defense mechanism, the unfolded protein response (UPR), a signaling cascade trying to restore ER homeostasis. However, if prolonged, unresolved ER stress leads to apoptosis. Unresolved ER stress contributes to the progressive death of pancreatic insulin-producing beta cells in both T1D and T2D. Diabetes mellitus is characterized by hyperglycemia, caused by the inability of the beta cells to maintain sufficient levels of circulating insulin. The current medications, insulin and antidiabetic drugs, alleviate diabetic symptoms but cannot reconstitute physiological insulin secretion which increases the risk of devastating vascular complications of the disease. Thus, one of the main strategies in improving current diabetes therapy is to define and validate novel approaches to protect beta cells from stress as well as activate their regeneration. Embryonic deletion of the *Manf* gene in mice led to gradual postnatal development of insulin-deficient diabetes caused by reduced beta cell proliferation and increased beta cell death due to increased and sustained ER stress. *In vitro*, recombinant MANF partly protected mouse and human beta cells from ER stress-induced beta cell death and potentiated mouse and human beta cell proliferation. Importantly, *in vivo* overexpression of MANF in the pancreas of T1D mice led to increased beta cell proliferation and decreased beta cell death, suggesting that MANF could be a new therapeutic candidate for beta cell protection and regeneration in diabetes.

## Introduction

Mesencephalic astrocyte-derived neurotrophic factor and its homolog CDNF form a group of unconventional neurotrophic factors with cytoprotective and regenerative effects for neurons and cardiomyocytes in different animal disease models (Lindholm et al., 2007; Airavaara et al., 2009, 2010; Voutilainen et al., 2009, 2011; Glembotski et al., 2012; Back et al., 2013; Yang et al., 2014; Cordero-Llana et al., 2015; Neves et al., 2016; Lindahl et al., 2017; Matlik et al., 2018). The exact function of MANF and CDNF in the nervous system is not yet understood but it clearly differs from traditional neurotrophic factors, neurotrophins and GDNF-family ligands which in neuronal development and maintenance perform their action through binding and activation of cell trans-membrane receptors leading to cell modulation, survival or death (Airaksinen and Saarma, 2002). MANF was identified as a small secreted protein with neuroprotective actions selective for dopamine neurons in culture (Petrova et al., 2003) and is commonly known for its ER stress regulated and regulating properties. *In vitro* MANF expression was upregulated and protective in primary cells and cell lines in response to chemically induced ER stress (Mizobuchi et al., 2007; Apostolou et al., 2008; Tadimalla et al., 2008; Hellman et al., 2011; Glembotski et al., 2012; Henderson et al., 2013). *In vivo*, MANF expression was increased in myocytes in a mouse model of myocardial infarction (Tadimalla et al., 2008; Glembotski et al., 2012). In rat brain, *Manf* mRNA levels were transiently increased after experimentally induced ischemia and status epilepticus (Lindholm et al., 2008). Chronic ER stress and disrupted ER homeostasis play a role in the pathogenesis of many diseases including neurodegenerative diseases, brain ischemia, DM (Lindholm et al., 2006; Szegezdi et al., 2006; Eizirik et al., 2008; Fonseca et al., 2011; Matlik et al., 2018), glomerular and tubular kidney disease (Inagi et al., 2014), and autoimmune diseases (Morito and Nagata, 2012). Thus, the mechanism behind the increased expression and protective effects of MANF in the different animal disease models is still not understood, but suggested to depend on its function in alleviating ER stress.

Recently, evidence for the role of MANF in modulating inflammation has emerged. MANF was shown to induce repair of damaged retina in flies and mice by alternative activation of innate M2-type immune cells toward protection (Neves et al., 2016). In addition, virus-delivered MANF-overexpression in the rat brain after cerebral ischemic injury promoted functional recovery by recruitment of phagocytic macrophages to the subcortical peri-infarct region indicating increased phagocytosis of myelin debris leading to faster recovery (Matlik et al., 2018). Thus, studies suggest that the MANF protective action could be mediated through activation of immune cells.

**FIGURE 1:**
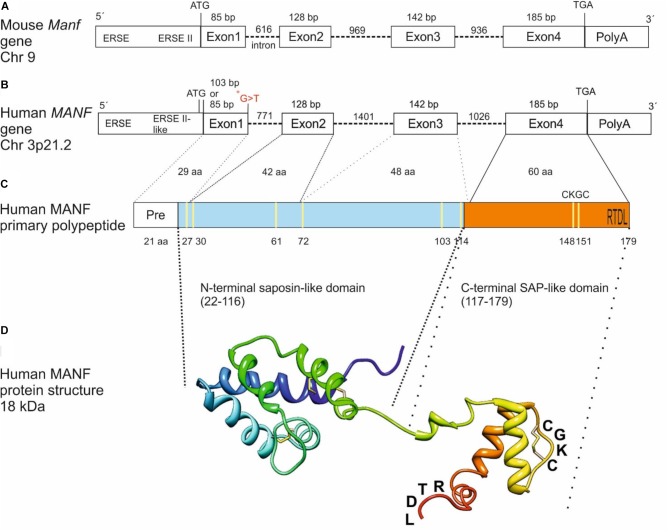
From gene to protein. Schematic structure of mouse **(A)** and human **(B)** *MANF* genes with 4 exons, primary polypeptide structure **(C)** and NMR solution structure of human MANF protein **(D)** with an N-terminal saposin-like domain (aa 22–116, light blue) and a C-terminal SAP-like domain (aa 117–179, orange). ^∗^G > T, homozygous missense mutation in a human patient with T2D (Yavarna et al., 2015) **(B)**. Cysteine residues are marked in yellow **(C)**. CXXC-motif and RTDL ER retention signal are indicated **(C,D)**. bp, base pair; Chr, chromosome; aa, amino acid; ERSE, ER stress-responsive element. Image in **(C)** modified from Lindahl et al. (2017) and in **(D)** from Hellman et al. (2011) and Lindstrom et al. (2013).

The mouse and human *MANF* genes are encoded by 4 exons generating a peptide of 179 amino acids with a signal sequence of 21 amino acids for secretion (**Figures 1A–C**) (Petrova et al., 2003; Lindholm et al., 2008). However, it is still unclear whether the human MANF signal sequence is 24 amino acids (UniProt database, Acc. No. P55145) instead of 21 as originally reported by Petrova et al. (2003). Based on amino acid sequence comparison, human MANF is 98% homologous with mouse (GenBank Acc. No. NP_083379) (Lindholm et al., 2008). MANF/CDNF are structurally distinct from classical neurotrophic factors and their amino-acid sequences with eight conserved cysteines forming four intramolecular disulfide bonds reveal no sequence homology with other proteins (**Figure 1C**) (Parkash et al., 2009; Hellman et al., 2011). Structure analysis of MANF and CDNF revealed two domain proteins with a N-terminal domain homologous to saposin-like proteins (SAPLIPs) (Parkash et al., 2009) and a carboxy(C)-terminal domain resembling the SAP-domain of Ku70, known to inhibit the proapoptotic activity of BAX (**Figures 1C,D**) (Sawada et al., 2003). The N-terminal saposin-like domain suggests binding to lipids and membranes whereas the C-terminal SAP domain proposes binding to DNA or to BAX inhibiting translocation of BAX to the mitochondria (Hellman et al., 2011). However, the anti-apoptotic effect of MANF in neurons seems not involve MANF binding to BAX (Matlik et al., 2015). The very C-terminal end of MANF contains a tetrapeptide RTDL sequence which resembles a typical ER retention motif, KDEL shared by several ER chaperons including GRP78/BiP (**Figures 1C,D**) (Raykhel et al., 2007; Capitani and Sallese, 2009). KDEL receptors are known to retro-transport chaperons with KDEL- or KDEL-like sequences, from the Golgi complex to the ER (Capitani and Sallese, 2009). In agreement, MANF has been found localized to the luminal ER in cell lines and neurons (Mizobuchi et al., 2007; Apostolou et al., 2008; Glembotski et al., 2012; Matlik et al., 2015). Consequently, MANF with mutated RTDL-sequence was found readily secreted from primary neurons and cell lines *in vitro* (Tadimalla et al., 2008; Glembotski et al., 2012; Henderson et al., 2013; Matlik et al., 2015; Oh-hashi et al., 2015).

MANF mRNA and protein are broadly expressed in neurons in the rodent nervous systems (Lindholm et al., 2008; Wang et al., 2014). However, it is also widely expressed in peripheral tissues with high expression in cells with elevated secretory functions (Lindholm et al., 2008; Lindahl et al., 2014).

Embryonic deletion of *Manf* in fruitfly (*Drosophila melanogaster*) demonstrated that MANF is important for the maturation of the nervous system as *Manf*-deletion led to early embryonic lethality, degeneration of axonal bundles in the central nervous system, lack of dopamine neurites and dramatic drop in dopamine levels (Palgi et al., 2009). Subsequently, knockdown of the *Manf* mRNA in zebrafish resulted in a significant decrease in the number of dopamine neurons in the diencephalic region suggesting that Manf is important for the differentiation and survival of dopaminergic neurons in zebrafish (Chen et al., 2012). The effect of MANF-deletion in the brain of MANF knockout mice was not as drastic as expected. MANF-deficiency in the embryonic mouse brain led to slower neurite extension upon neuronal differentiation, which was caused by reduced *de novo* protein synthesis and activated UPR (Tseng et al., 2017). In addition, MANF-deficiency led to increased neuronal vulnerability to hypoxia/reperfusion as the brain infarction volume was significantly increased in experimentally induced focal cerebral ischemia in neuron-specific MANF-deficient mice (Matlik et al., 2018).

Despite the protecting and restorative effects of MANF for many cell types and organs in mammals, important roles for MANF in pancreatic beta cells have emerged. MANF-deficient (*Manf^-/-^*) mice developed insulin-deficient DM due to progressive postnatal decrease in beta cell proliferation and increase in beta cell death (Lindahl et al., 2014). One of the reasons behind reduced beta cell mass was found to be chronic irreversible ER stress. Furthermore, recombinant MANF protein was able to induce primary mouse and human beta cell proliferation in culture and to partly protect human and mouse beta cells from ER stress-induced beta cell death (Lindahl et al., 2014; Cunha et al., 2017; Hakonen et al., 2018). Modulation of inflammatory NF-κB signaling was suggested as one of the protective mechanism behind MANF action (Hakonen et al., 2018). *In vivo*, virus-delivered MANF-overexpression increased beta cell proliferation and rescued beta cells from streptozotocin-induced beta cell death in the pancreas of T1 diabetic mice (Lindahl et al., 2014). The emerging protective and regenerative effects for MANF in mouse and human pancreatic beta cells and diabetes are discussed in this review.

## Diabetes Mellitus

Diabetes mellitus is a major endocrine metabolic disorder characterized by hyperglycemia caused by either insulin deficiency and/or insulin resistance. Poorly controlled diabetes may lead to severe long-term complications, including microvascular (including retinopathy, nephropathy and neuropathy) and macrovascular (including atherosclerosis and amputation) diseases leading to disability and death. DM affects more than 425 million people worldwide (International Diabetes Federation [IDF], 2017; Diabetes Atlas) and is rapidly increasing. Typically, diabetes is classified into two major subtypes, T1D and T2D where T1D diabetes is caused by the progressive autoimmune destruction of pancreatic beta cells (Pirot et al., 2008). The pancreas is a mixed gland consisting of exocrine tissue with acinar cells important for production and secretion of digestive enzymes and endocrine Langerhans islets where majority of cells (70–80%) are insulin-producing beta cells (Cabrera et al., 2006; Murtaugh, 2007). Beta cells are responsible for the synthesis, storage and release of insulin, which is a key anabolic hormone in the regulation of metabolism. At the time of T1D diagnosis, about 70–80% of the beta cells are lost (Cnop et al., 2005). Both environmental, such as viral infections, drugs and dietary factors during infancy, as well as genetic factors, contribute to the development of T1D (Pirot et al., 2008). Several susceptibility loci for T1D have been found and diabetes-predictive circulating autoantibodies against beta cell antigens such as insulin can be found in non-diabetic subjects years before clinical manifestation of the disease. The process of beta cell death in T1D is not fully understood. However, the first step involves T lymphocytes recognizing self-antigens in the lymph nodes (Wan et al., 2018), resulting in cytotoxic activated T lymphocytes invading islets and killing beta cells through the extrinsic death pathway via death-receptor Fas pathway and granzyme/perforin system (Pirot et al., 2008). The inflammatory cascade also involves macrophages, dendritic cells, T and B lymphocytes invading the islets resulting in insulitis and beta cell death through the action of cytokines and chemokines (Pirot et al., 2008). In combination with increased ER stress, production of nitric oxide (NO), activation of JNK inflammatory pathways, mitochondrial dysfunction and production of free radical this leads to execution of the intrinsic or mitochondrial beta cell death pathway (Eizirik et al., 2008).

T2D is characterized by hyperglycemia caused by insulin resistance in peripheral tissues and defects in insulin secretion, metabolic dysfunction and death of pancreatic beta cells (Kolb and Eizirik, 2011). Insulin resistance is likely caused by modern Western life style with diet rich in saturated fat, minimal exercise, and shortened duration of sleep, resulting in obesity but also by genetic predisposition (Kolb and Mandrup-Poulsen, 2010; Donath and Shoelson, 2011). T2D develops when the islet beta cells cannot compensate for the degree of insulin resistance leading to metabolic stress including mitochondrial oxidative and ER stress activating the UPR, insulin deficiency and chronic hyperglycemia and beta cell dysfunction and death. The dysfunction of beta cells and increased beta cell death in T2D patients are likely caused by nutrient-induced systemic subclinical inflammation and as a hormetic response trying to rescue beta cells from death (Cnop et al., 2005; Donath and Shoelson, 2011; Kolb and Eizirik, 2011).

The current medications, insulin and anti-diabetic drugs, can transiently decrease the hyperglycemia in diabetic patients and rather represent ameliorative treatment than cure. Thus, their administration does not truly mirror the physiological response of beta cells and therefore does not prevent the devastating micro- and macrovascular complications of the disease. The reasons for beta cell destruction are not clear but there are evidence suggesting prolonged ER stress and chronic activation of the UPR as one common pathogenic mechanism for beta cell dysfunction and death in both T1 and T2D (Eizirik et al., 2008; Fonseca et al., 2011; Cnop et al., 2012).

## ER Stress and UPR Signaling in Beta Cells

The ER directs the folding, processing and trafficking of membrane bound and secreted proteins. A number of genetic and environmental insults result in ER stress, which is caused by the accumulation and aggregation of unfolded or misfolded proteins in the ER (Wang and Kaufman, 2016). Beta cells contain a large and well-developed ER, specialized for insulin biosynthesis and its secretion. Beta cells are under normal conditions continuously exposed to low threshold levels of UPR signaling playing major roles in normal ER biogenesis and expansion of the ER network (Sriburi et al., 2007; Bommiasamy et al., 2009). Increased insulin demand leads to accumulation of misfolded proinsulin which results in ER stress activating UPR signaling pathways in beta cells. This leads to temporarily reduced protein synthesis to prevent further accumulation of misfolded proteins, recovery of protein folding, clearance of misfolded and unfolded proteins by the ERAD pathway involving the cytoplasmic ubiquitin-proteasome system and activation of autophagy to promote clearance (Ron and Walter, 2007; Cnop et al., 2017). Actually, pseudotime RNA sequencing analysis of single non-diabetic human beta cells revealed transition between states of high activity by insulin expression and low UPR, and states of low insulin expression and high expression of UPR markers, including the master regulator of antioxidant response NRF2 (Xin et al., 2018). Interestingly, MANF was upregulated along with other UPR genes in this state and has previously been suggested to promote the activation of NRF2 (Zhang et al., 2017; Xin et al., 2018). This UPR-mediated stress recovery state with low rates of insulin biosynthesis was also enriched for proliferating beta cells (Xin et al., 2018).

The UPR signaling is mediated via three major ER transmembrane sensors, protein kinase RNA(PKR)-like ER endoplasmic reticulum kinase (PERK), endoribonuclease inositol-requiring protein 1 (IRE1 α) and activating transcription factor 6 (ATF6 α and β) (Wang and Kaufman, 2016). The activation of these sensors leads to downstream signaling cascades aiming to reduce protein synthesis, increase transcription of genes involved in global proteostasis control and increase the degradation of misfolded proteins. If ER stress is unresolved, UPR shift from an adaptive (A-UPR) response to a chronic unresolved UPR leading toward increased inflammatory signaling, autophagy, terminal UPR (T-UPR) and apoptosis (Tabas and Ron, 2011; Hetz and Papa, 2018). Under normal conditions ER chaperone GRP78, binds the luminal domain of PERK and IRE1α through its ATPase domain keeping the sensors inactive (Carrara et al., 2015). However, when unfolded proteins are accumulated in the ER, GRP78 associates with the unfolded proteins and releases the sensors leading to IRE1α and PERK dimerization or oligomerization and trans-autophosphorylation. GRP78 releases ATF6 for translocation to the Golgi where it is cleaved into functional transcription factor ATF6f. PERK phosphorylates eukaryotic translation initiator factor 2α (eIF2α) at serine 51 which reduces overall translation but selectively allows translation of ATF4 and other proteins thus contributing to reduction of the polypeptide load and increasing the folding capacity at the ER. ATF4 regulates genes involved in amino acid biosynthesis, amino acid transport and anti-oxidative response (Schroder and Kaufman, 2006). Insulin has been found to regulate ATF4 expression (Malmberg and Adams, 2008). In chronic ER stress, sustained ATF4 translation contributes to the induction of apoptosis through inducing expression of pro-apoptotic CHOP (Harding et al., 2000). CHOP directly promotes the growth arrest and DNA damage protein GADD34, which dephosphorylates eIF2α via PP1 and leads to protein translation recovery and feed-back attenuating ER stress. In sustained ER stress, in cooperation with ATF4, CHOP triggers increase in pro-apoptotic protein production, cellular ATP depletion, formation of ROS and oxidative stress contributing to the cell death (Han et al., 2013). Additionally, CHOP promotes cell death by inhibiting the expression of anti-apoptotic BCL-2 and by inducing the pseudokinase tribbles homolog 3 (*TRIB3)* expression (McCullough et al., 2001; Ohoka et al., 2005). TRIB3 is an inhibitor of AKT in the insulin signaling pathway and its expression is also regulated by ATF4 (Cunard, 2013). In addition *Trib3* was increased after thapsigargin-induced ER stress in rodent beta cells and overexpression of TRIB3 led to beta cell death by activation of the NF-κB signaling pathway (Fang et al., 2014), thus indicating that TRIB3 is an important factor in ER stress-induced beta cell failure. Furthermore, eIF2α phosphorylation can promote the activation of pro-inflammatory NF-κB signaling pathway through the rapid reduction in its inhibitor (IκBα) due to general translation inhibition (Deng et al., 2004).

Conformational changes in trans-autophopshorylated IRE1α leads to activation of IRE1α RNase activity which catalyzes the removal of a 26 bp intron from the unspliced XBP1u mRNA generating when translated, an active transcription factor XBP1s. XBP1s induces the upregulation of ER chaperones, components of the ERAD machinery and phospholipid biosynthesis. The RNase activity in IRE1α also regulates the cleavage or decay of multiple RNAs including insulin in pancreatic beta cells (Lipson et al., 2008). The regulated IRE1α-dependent decay or RIDD has thus important physiological functions in decreasing the ER workload for newly synthesized proteins especially in cells with high secretory function (Coelho and Domingos, 2014). Hyperactive RNase activity of IRE1α leads to upregulation of caspase-2 and pro-oxidant TXNIP important for the T-UPR leading to beta cell apoptosis and diabetes via the intrinsic/mitochondrial death pathway (Lerner et al., 2012). In chronic ER stress, hyperoligomerized IRE1α recruits TRAF2 by its cytosolic domain and activates ASK1 at the ER membrane (Urano et al., 2000; Nishitoh et al., 2002; Brozzi and Eizirik, 2016). IRE1α/TRAF2/ASK1 complex triggers apoptosis via p38 MAPK and JNK pathways followed by enhanced transcriptional activity of activator protein 1 (AP-1) dependent pro-inflammatory genes (Urano et al., 2000; Nishitoh et al., 2002; Kim et al., 2010). Finally, IRE1α/TRAF2 complex also activates pro-inflammatory NF-κB signaling pathway via IκBα proteosomal degradation by ERAD (Kaneko et al., 2003), followed by expression of inducible isoform nitric oxide synthases (iNOS) and subsequent nitric oxide (NO) formation. NO provokes beta cell death through oxidative stress, disrupted energy metabolism, DNA damage via necrosis pathway mediated by activation of poly(ADP-ribose) polymerase (PARP) or depletion of ER Ca^2+^ (Oyadomari et al., 2001; Cardozo et al., 2005; Meares et al., 2013).

ER-stress mediated activation of ATF6 leads to its translocation to Golgi, where it is cleaved by site 1 and 2 proteases (S1P) and (S2P). The cleaved form of ATF6 is a transcriptional activator of chaperones GRP78 and GRP94, CHOP, Xbp1s, ERAD target genes and genes involved in lipid biosynthesis (Kaufman et al., 2010). ATF6α and ATF6β recognize conserved ER stress regulatory elements but have different transcriptional activation domains suggesting that the relative levels of ATF6α and ATF6β may regulate the duration and strength of ATF6-dependent gene expression and cell viability (Thuerauf et al., 2007). Prolonged activation of ATF6 pathway in the beta cells restrains insulin gene expression and results in beta cell dysfunction and death (Seo et al., 2008).

## ER Stress-Related Mechanisms of Beta Cell Death in T1D

In T1D the inflammatory response caused by the release and binding of cytokines such as TNF-α, IL-1β, and IFN-γ to receptors on the beta cell membrane, trigger apoptosis via activation of mitochondrial intrinsic pathway involving BCL2 family of anti-apoptotic and pro-apoptotic proteins (Gurzov and Eizirik, 2011). Death effectors (e.g., BAX, BAK) trigger the loss of mitochondrial membrane integrity followed by release of cytochrome *c*, increase in intracellular calcium and mitochondrial ROS production (Gurzov and Eizirik, 2011). Cytochrome *c* released from mitochondria binds to APAF-1, which forms the apoptosome together with ATP and procaspase-9, resulting in caspase-9 followed by caspase-3 activation and beta cell death. The cell death followed after chronic ER stress has been suggested to involve activation of BH3 only proteins DP5 and PUMA (Gurzov and Eizirik, 2011).

There is a strong link between the chronic activation of the UPR, especially the IRE1α and PERK pathways, and cytokine induced beta cell death in T1D (Brozzi and Eizirik, 2016). Pro-inflammatory cytokines cause beta cell death also trough ER stress leading to activation of the pro-apoptotic JNK, p38 and inflammatory NF-κB pathway (Urano et al., 2000; Cardozo et al., 2005; Brozzi and Eizirik, 2016). In rat beta cells IL-1β and IFNγ induce NO production and consequent SERCA2b inhibition, leading to ER Ca^2+^ depletion and activation of IRE1α, increased levels of phosphorylated JNK, CHOP expression, and beta cell death (Cardozo et al., 2005; Brozzi and Eizirik, 2016). In contrast, in human and mouse beta cells, PERK and IRE1α pathways are activated independent of increased NO production (Brozzi and Eizirik, 2016).

Sustained ER stress is one of the potential cause leading to beta cell death in human patients with T1D and T1D mouse models (Eizirik et al., 2008; Cnop et al., 2017). Increased levels of ATF3, CHOP and GRP78 were found in inflamed islets of T1D patients (Hartman et al., 2004; Marhfour et al., 2012). NOD spontaneously develop diabetes and is a commonly used mouse model for T1D (Anderson and Bluestone, 2005). First signs of insulitis occurs in NOD mice already at 3 weeks of age (Fujino-Kurihara et al., 1985) and as disease progresses, the beta cell mass decreases and hyperglycemia occurs in female mice between 12 and 20 weeks of age, indicating the onset of diabetes. Importantly, increased ER stress has been found to precede the development of beta cell failure and diabetes in NOD mice, which is accompanied by increased expression of NF-κB target genes (Tersey et al., 2012). UPR target genes *Grp78, Xbp1s* and *Chop*, were also upregulated while *Atf4* mRNA levels were decreased before the downregulation of beta cell markers at the age of 6 weeks in pre-diabetic NOD mice (Tersey et al., 2012; Morita et al., 2017). Interestingly, another study detected upregulation of *Manf* mRNA along with increased auto-phosphorylation of IRE1α in islets of pre-diabetic NOD mice (Morita et al., 2017). Additionally, decreased expression of *Serca2b* mRNA was detected in pre-diabetic NOD islets, indicating impaired ER Ca^2+^ homeostasis (Tersey et al., 2012).

The Ins2^Akita^ mouse is a mutant model of T1D, which expresses the proinsulin variant gene, *Ins2* (C96Y) and heterozygous Ins2^AKITA^ mice develop hyperglycemia and hypoinsulinemia due to beta cell death (Wang et al., 1999). The Ins2^Akita^ mutation disrupts a disulfide bond between insulin chain A and B thus leading to a conformational change in the protein. Accumulation of the misfolded proinsulin and blocking trafficking to the Golgi led to increased ER stress and upregulated UPR markers, *Grp78* and *Chop* mRNA (Wang et al., 1999; Araki et al., 2003). Additionally, beta cells in Akita mice contained secretory insulin granules of reduced number and size, accompanied with dilated ER, which is a hallmark for ER stress and dysfunction (Wang et al., 1999). Importantly, MANF protein was upregulated in pancreatic beta cells of Akita mice probably due to the ER stress (Mizobuchi et al., 2007).

## ER Stress and T2D

In T2D, beta cells are exposed to local environmental factors such as glucolipotoxicity and inflammatory cytokines leading to increased insulin and ceramide production, ER- and oxidative stress and intrinsic beta cell death (Cnop et al., 2010, 2012). Increasing levels of saturated FFAs such as palmitate activate all three UPR branches in beta cells shown both *in vitro* and *in vivo*, leading to beta cell dysfunction and death (Cunha et al., 2008; Cnop et al., 2010, 2012). *In vitro* exposure of human islets to palmitate led to ER stress followed by NF-κB activation and inflammatory responses (Igoillo-Esteve et al., 2010). In pancreatic islets from T2D patients, increased activation of classical UPR-induced proteins p58^IPK^, CHOP, GRP78, ATF3 and distended ER were found (Hartman et al., 2004; Marchetti et al., 2004; Huang et al., 2007; Laybutt et al., 2007). However, decreased expression levels of ATF6, XBP1s and almost no eIF2α phosphorylation were detected in the islets of long term T2D patients (Engin et al., 2014), suggesting that deficient activation of the UPR leads to beta cell demise in long term patients.

*In vivo*, islets from animals fed with high fat diet or from genetic T2D mouse or rat models showed increased ER stress in beta cells (Cnop et al., 2012). The leptin receptor deficient db/db mice develop hyperinsulinemia and obesity already a few weeks after birth. Hyperglycemia appears at the age of 8 weeks followed by beta cell dysfunction (Dalboge et al., 2013). Increased beta cell mass occurs in young animals (10 weeks of age) whereas beta cell mass declines in older animals (Dalboge et al., 2013). Enhanced expression of phosphorylated eIF2α, increased *Xbp1s* and *Chop* mRNA were observed in the islets of insulin resistant db/db mice at the age of 10–12 weeks (Yusta et al., 2006; Laybutt et al., 2007). Another study showed significant upregulation of *Grp78, p58* and *Grp94* mRNA as well as increased expression of UPR genes (*Atf3, Chop*, and *Trib*3) in islets from prediabetic *db/db* mice (Chan et al., 2013), strongly indicating the involvement of chronic increased ER stress in the beta cells of T2D diabetic mice. Along with upregulated UPR genes, RNA sequencing analysis revealed increased *Manf* mRNA levels in beta cells of 12-week-old db/db mice (Neelankal John et al., 2018), suggesting that MANF contributes in UPR signaling also in T2D beta cells.

Glucotoxicity, causing high ER- and oxidative stress in T2D beta cells, has been implicated to lead to dedifferentiation of beta cells. Cells reversibly lose their beta cell identity through downregulation of beta cell specific transcriptional genes such as *Pdx1, MafA, Nkx6.1*, and *Pax6*, and even gaining features of other pancreatic endocrine cell types such as glucagon-producing alpha cells (Weir et al., 2013; Szabat et al., 2016; Swisa et al., 2017). In contrast, in normal conditions, little evidence of apoptosis and dedifferentiation of human beta cells have been found (Xin et al., 2018). In glucotoxic conditions beta cells, but also islet alpha-cells seem to suffer from chronic ER stress, with the exception that alpha cells are more resistant to ER stress-induced apoptosis (Marroqui et al., 2015). Acute and chronic glucose stimulation causes activation of IRE1α/Xbp1 pathway in pancreatic mouse and rat beta cells *in vitro* (Lipson et al., 2006; Elouil et al., 2007). In addition, chronic hyperglycemia increases proinsulin biosynthesis and formation of IAPP in the beta cells which leads to accumulation of misfolded IAPP and ROS production, disruption of ER Ca^2+^ homeostasis, activation of the UPR pathways leading to proinsulin degradation and beta cell death (Haataja et al., 2008; Hasnain et al., 2016).

## Dysregulated UPR Signaling and Diabetes in Human and Mice

Genetic mutations in various UPR components cause inherited syndromes and diabetes both in rodents and humans. Recessive homozygote mutations in the eIF2α kinase domain of the PERK (EIF2AK3) gene results in Wolcott-Rallison syndrome, characterized by infantile non-autoimmune insulin-dependent diabetes, multiple epiphyseal dysplasia, defects in exocrine pancreas, hepatic steatosis, microcephaly, intellectual disability and growth retardation (Delepine et al., 2000; Senee et al., 2004). Similarly to humans, homozygous ablation of the *Perk* gene (*Perk^-/-^)* in mice recapitulates the defects of the human syndrome, demonstrating loss of insulin-secreting beta cells and development of diabetes, followed by the loss of glucagon-secreting alpha cells and failure of the exocrine pancreas, skeletal dysplasias followed by postnatal growth retardation (Harding et al., 2001; Zhang et al., 2002). Recent studies demonstrated that PERK deletion from the pancreases of young and mature adult mice resulted in hyperglycemia associated with loss of beta cells and islet architecture, indicating the importance of PERK in adult pancreas for maintaining glucose homeostasis (Gao et al., 2012). Activation of the IRE1α and ATF6 UPR branches led to increase in pro-apoptotic JNK signaling in PERK-excised islets (Gao et al., 2012). In contrast, homozygous knock-in mutation at the PERK phosphorylation site in eIF2α (Ser51Ala) in mice lead to a neonatal lethal phenotype, characterized by defects in embryonic beta cell survival (Scheuner et al., 2001). Further studies performed on heterozygous eIF2α mice revealed insulin resistance with elevated fasting blood glucose levels, defects in beta cell function and glucose intolerance after the feeding with high fat diet due to accumulation of misfolded proinsulin (Scheuner et al., 2005). Interestingly, ATF4 deficient mice did not show any signs of beta cell impairment, although the ATF4 responses were abolished in diabetic *Perk^-/-^* mice (Back et al., 2009).

Wolfram syndrome 1, is a rare autosomal-recessive neurodegenerative disease caused by mutation in the Wolframin 1 gene (WFS1) and characterized by juvenile-onset insulin-dependent diabetes, neurodegenerative disorder, optic atrophy, hearing impairment, and psychiatric illness (Cryns et al., 2003). Common polymorphisms in the *WFS1* gene have been associated with T2D (Sandhu et al., 2007; Franks et al., 2008). The *WFS1* gene encodes for a transmembrane ER protein and its expression is induced by increased ER stress (Fonseca et al., 2005). Wolframin is highly expressed in pancreatic beta cells and it has been shown to negatively regulate the expression of ATF6α target genes and enhance ATF6α ubiquitin-mediated proteosomal degradation thus also attenuating *Grp78* and *Xbp1* mRNA expression (Fonseca et al., 2010). In agreement, deficient *WFS1* expression in mice and human led to hyperactivation of the ATF6α pathway, and subsequent increased activation of the ATF4, CHOP and IRE1α/XBP1 pathways inducing beta cell dysfunction and apoptosis resulting in diabetes (Ishihara et al., 2004; Riggs et al., 2005; Yamada et al., 2006; Fonseca et al., 2010; Shang et al., 2014).

Inactivation of *Ire1α* in mice resulted in widespread developmental defects and embryonic lethality (Zhang et al., 2005). Embryonic inactivation of the *Ire1α* gene specifically in beta cells in mice resulted in a diabetic phenotype including impaired glycemic control and defects in insulin biosynthesis and secretion postnatally due to decreased oxidative folding of proinsulin along with decreased expression of protein disulfide isomerases (Tsuchiya et al., 2018). Deletion of *Ire1α* specifically from beta cells in adult mice lead to hyperglycemia and hypoinsulinemia, due to impaired beta cell function, which was exacerbated upon high fat diet feeding and glucose stimulation (Hassler et al., 2015). Moreover, deletion of IRE1α from beta cells as well as hypothalamus in mice resulted in obesity and insulin resistance, when kept on a high-fat diet (Xu et al., 2014). Conditional ablation of *Xbp1* specifically from the beta cells resulted in impaired ER homeostasis associated with diminished proinsulin processing, reduced insulin secretion and cell proliferation due to hyperactivation of IRE1α (Lee et al., 2011).

Similarly, to IRE1α knockout mice, simultaneous embryonic ablation of *ATF6*α and *ATF6*β in mice lead to embryonic lethality in the early developmental stage with severe developmental defects (Wu et al., 2007; Yamamoto et al., 2007). However, neither beta cell development nor function were affected by removal of ATF6α specifically from beta cells (Engin et al., 2013). However, when carrying the Ins2^Akita^ allele or under high fat diet *Atf6α-*deficient mice displayed decreased insulin secretion (Usui et al., 2012), indicating that ATF6α contributes to both beta cell survival and beta cell death.

Thus deficiency in any of the key proteins in the UPR pathway seem to lead to dysregulated UPR response, defect in beta cell function or death in human and mice.

## MANF-Deficiency in Beta Cells Leads to Sustained ER Stress, Reduced Proliferation and Beta Cell Death

**FIGURE 2:**
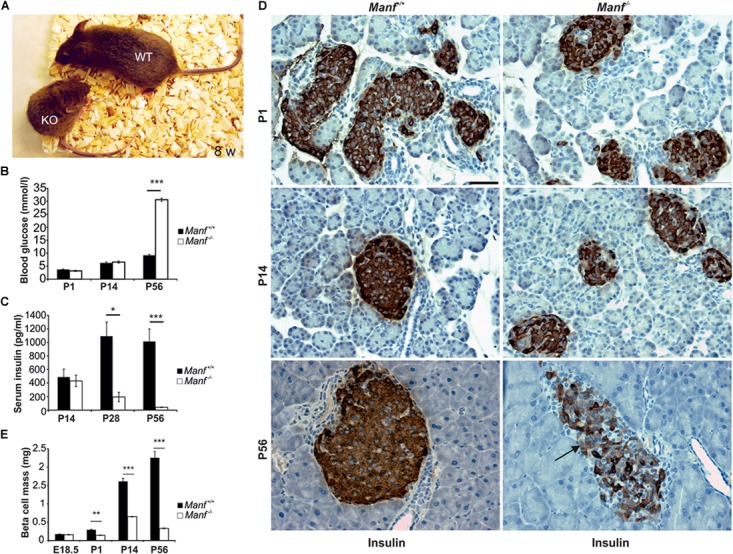
MANF-deficient mice develop insulin-dependent diabetes caused by progressive decrease in beta cell mass. **(A)** *Manf^-/-^* mice at 8 weeks of age show a severe growth defect compared to WT littermates. **(B,C)** *Manf^-/-^* mice develop diabetes, characterized by increased blood glucose levels **(B)** and decreased serum insulin levels **(C). (D)** Histological analysis revealed reduced insulin staining, reduced islet size and number of beta cells in pancreases of newborn, 2 and 8 weeks old *Manf^-/-^* pancreases. **(E)** Progressive loss of beta cell mass in *Manf^-/-^* mice compared to controls. Images in **(B–E)** modified from Lindahl et al. (2014). Scale bar, 50 μm.

To understand the physiological roles for MANF in mammals we created MANF conventional knockout (*Manf^-/-^)* mice where *Manf* mRNA expression was disrupted by efficient splicing of exon 2 to a β-galactosidase reporter gene (Lindahl et al., 2014). The *Manf^-/-^* mice showed poor survival after birth, a severe growth retardation and died at 2–3 months of age (**Figure 2A**). Unexpectedly, homozygote *Manf^-/-^* mice developed insulin-dependent diabetes after birth (**Figures 2B,C**) due to progressive decrease in beta cell proliferation and increased beta cell death. The beta cell mass in *Manf^-/-^* mice was decreased by 50% already at P1 (**Figures 2D,E**), whereas the glucagon-producing alpha cell mass remained unchanged compared to wild-type litter-mate mice. Insulin positive beta cells were barely detectable in 8-week-old knockout pancreases (**Figure 2D**).

The function of MANF in humans, is not known. A clinical exome sequencing study of patients with neurocognitive disorders in a highly consanguineous Middle Eastern population revealed a young 22-year-old patient with a homozygous missense mutation in the splice donor site in exon 1 of the human MANF gene (**Figure 1B**) (Yavarna et al., 2015). This mutation most likely is the cause of obesity, T2D, short stature, mild intellectual disability, microcephaly, hypothyroidism, hypogonadism, myopia and autoimmune alopecia. However, nothing is known about the actual MANF mRNA and protein levels in this patient. Nevertheless, similarities with short stature and diabetes was found in *Manf^-/-^* mice (Lindahl et al., 2014).

## Chronic Activation of the Unfolded Protein Response Leads to Beta Cell Demise in *Manf^-/-^* Mice

The mammalian MANF promoter contains ER stress responsive elements ERSE and ERSEII, which are important for XBP1s and ATF6 binding leading to upregulation of MANF expression in response to ER stress (**Figure 1A**) (Mizobuchi et al., 2007; Oh-Hashi et al., 2013; Wang et al., 2018). In a recent genome-scale CRISPR-interference screen, MANF was identified as a gene whose repression perturbs ER homeostasis (Adamson et al., 2016). In single cells deleted for different combinations of UPR genes, ATF6α but not PERK or IRE1α was shown to be required for the upregulation of MANF expression in ER stress conditions induced by tunicamycin or thapsigargin (Adamson et al., 2016). This confirms the importance of ATF6α as a transcription factor for MANF in ER stress (Adamson et al., 2016). Importantly, MANF was shown to bind GRP78 in a Ca^2+^-dependent manner in cardiomyocytes and HeLa cells and this binding was abolished, and MANF was secreted from cells when Ca^2+^ was depleted from the ER by thapsigargin (Glembotski et al., 2012).

Consequently, ER stress levels and activation of the UPR pathways was studied as one of the mechanisms behind the beta cell demise in *Manf^-/-^* mice. Increased *Chop* and *Xbp1s* mRNA expression in the IRE1α pathway was found already in the embryonic *Manf^-/-^* pancreases (Lindahl et al., 2014). As the expression levels of beta cell specific markers *Glut2, Insulin1/2* and *Pdx1*, were similar in the embryonic pancreases of WT and *Manf^-/-^* mice but significantly reduced after birth in *Manf^-/-^* islets, ER stress was suggested to precede the reduced beta cell phenotype in *Manf^-/-^* islets. In islets of P1 and 2-week-old *Manf^-/-^* mice UPR genes in the PERK and ATF6α pathways were upregulated and eIF2α phosphorylated confirming sustained UPR activation in the MANF-deficient beta cells. MANF-deficiency in beta cells was suggested to lead to prolonged ER stress resulting in reduced beta cell phenotype, decreased beta cell proliferation and increased beta cell death. In accordance, maternal and zygotic deletion of *Manf* in fruitfly led to embryonic lethality and increased expression of UPR genes (Palgi et al., 2009, 2012).

As MANF is widely expressed in other tissues, the question remains why *Manf^-/-^* mice have such a robust beta cell specific phenotype? Beta cells express high levels of proteins involved in UPR compared to cells in other tissues, suggesting that beta cells are adapted to signal ER stress (Cnop et al., 2017). However, beta cell dysfunction and death shown by deficient expression of UPR genes in humans with diabetes and animal models, indicate that beta cells are extremely sensitive to the dysregulation of ER stress.

How would chronic UPR activation decrease the expression of beta cell specific gene? Attenuation of global mRNA translation by prolonged eIF2α phosphorylation is known to be poorly tolerated by beta cells (Cnop et al., 2007). In addition, global decrease in translation reduces expression of beta cell specific genes as well. Prolonged activation of IRE1α has been shown to lead to RIDD induced insulin mRNA degradation (Lipson et al., 2008), whereas prolonged expression of ATF6 has been shown to downregulate the expression of *Pdx1* and *MafA*, both important for insulin expression (Seo et al., 2008; Artner et al., 2010).

The beta cell mass is expanded during early embryonic pancreas development through differentiation of a population of progenitor cells (neogenesis) and proliferation (Dhawan et al., 2007). During late embryogenesis and in young rodents the beta cell mass is mainly expanding through extensive proliferation which declines rapidly with age (Teta et al., 2005). The beta cells are long-lived and in adult rodents the major mechanism of slow-turnover beta cell replacement is by replication of pre-existing beta cells (Dor et al., 2004). The reason for decline in beta cell proliferation in newborn and young *Manf^-/-^* mice compared to WT was likely at least in part due to the prolonged UPR and phosphorylation of eIF2α leading to translational arrest (Lindahl et al., 2014). It has been shown that global decrease in mRNA translation due to sustained phosphorylation of eIF2α resulted in cell cycle arrest at G_1_ phase (Brewer and Diehl, 2000). In a recent study genetically reduced insulin production in mice led to reduced PERK/eIF2α and UPR signaling in pancreatic beta cells, thus reducing ATF4 translation and consequent expression of target gene *Trib3* (Szabat et al., 2016). TRIB3 has been shown to disrupt insulin-signaling in liver cells by directly binding and blocking the kinase activity of AKT (Du et al., 2003). Thus, decreased ER stress in beta cells expressing lower levels of insulin was suggested to promote proliferation through the activation of the AKT-Cyclin D1 axis. This would suggest that the decreased beta cell proliferation in *Manf^-/-^* islets could be triggered by the increased PERK/EIF2α activation, leading to increased *Trib3* expression thus blocking AKT-cyclinD1 activity.

A recent study revealed that genetically induced MANF overexpression in the hypothalamus of mice led to increased feeding behavior and obesity, impaired insulin signaling and insulin-resistance in the hypothalamus, whereas decreased MANF-expression in the hypothalamus resulted in reduced feeding behavior and body weight (Yang et al., 2017). Overexpressed MANF was suggested to recruit and activate PIP4k2b in the ER, thus reducing AKT phosphorylation downstream of insulin receptor signaling leading to hyperphagia and obesity (Yang et al., 2017).

The above studies might suggest that MANF levels are critical for modulating insulin signaling in different cell types by (1) regulating ER stress levels and thus downstream TRIB3 expression and (2) by interacting with PIP4k2b in the ER and thus regulating AKT phosphorylation.

## MANF Expression in Mouse and Human Pancreatic Beta Cells

MANF was found to be highly expressed in mouse beta cells as well as in pancreatic exocrine acinar cells (Lindahl et al., 2014). In humans, MANF was highly expressed already in embryonic pancreatic epithelium and in embryonic cells double positive for glucagon and insulin (Hakonen et al., 2018). In adult human pancreases MANF was expressed in the exocrine tissue and in islet beta cells but not in glucagon-producing alpha cells or pancreatic polypeptide cells (Hakonen et al., 2018). The lack of MANF protein in alpha cells supports our finding that the alpha cell mass was not affected in the *Manf^-/-^* islets (Lindahl et al., 2014) and thus MANF seems not to be needed for the survival of alpha cells. In EndoC-βH1 cell line, which is a valid model of human beta cells (Tsonkova et al., 2018), MANF was co-localized with protein disulfide isomerase (PDI) in the ER confirming that MANF is localized to the ER also in beta cells (Hakonen et al., 2018).

## ER Stress-Induced MANF Secretion

The expression profiles of genes differently regulated in T1D beta cells and human beta cells exposed to pro-inflammatory cytokines *in vitro* are very similar (Eizirik et al., 2012; Lundberg et al., 2016). EndoC-βH1 cells and primary beta cells are sensitive to cytokine-induced ER stress, thus partly modeling the mechanism of beta cell death in T1D *in vitro* (Cardozo et al., 2005; Eizirik et al., 2008; Tsonkova et al., 2018). In agreement with this, increased levels of UPR markers, both in human islets and EndoC-βH1 cells were found in response to cytokines in culture (Hakonen et al., 2018). In addition, increased MANF expression was detected in human islets and EndoC-βH1 cells exposed to cytokine cocktail IL-1β, IFN-γ, IL-17 and TNF-α in culture. A progressive increase in MANF secretion from cytokine-treated EndoC-βH1 cells was also detected. Importantly, MANF was found to be secreted independently from insulin, as MANF secretion was not affected in KCl-depolarized EndoC-βH1 cells induced to secrete insulin (Hakonen et al., 2018). Thus, MANF seems to be secreted from human beta cells in response to ER stress but the precise mechanism of MANF secretion from human and rodent beta cells are to be investigated. *In vitro*, MANF has been shown to be constitutively secreted but its secretion was highly increased by chemical compounds inhibiting SERCA ATPase pump thus depleting Ca^2+^ from the ER in cardiomyotes and HeLa cells (Glembotski et al., 2012). *In vivo*, secretion of MANF was increased by ER stress in chondrodysplasias and ER-stress induced kidney diseases (Hartley et al., 2013; Kim et al., 2016, 2017), suggesting that high ER stress increases MANF secretion in pathological conditions.

We have previously shown that MANF protein levels are increased in sera from young children newly diagnosed with T1D (Galli et al., 2016). The highest MANF levels were found in sera from children closest to diagnosis. Similarly, serum MANF levels were increased in patients diagnosed with prediabetes and T2D, correlating with indexes of insulin resistance (Wu et al., 2017). However, it is intriguing from where the increased MANF levels are derived. After initiation of insulin therapy in T1D the lowering of blood glucose levels and insulin demand leads to decreased ER stress and increased insulin production in the remaining beta cells known as the “honeymoon” period in T1D (Clark and Urano, 2016). During this period MANF might be upregulated and secreted from beta cells in order trying to rescue the remaining beta cells. The beta cells in newly diagnosed T1D patients may have dedifferentiated upon hyperglycemia as suggested by some studies (Weir et al., 2013), and could therefore resume beta cell phenotype upon insulin treatment and normoglycemia. MANF expression may thus be upregulated and secreted shown as the increased MANF levels in sera of newly diagnosed T1D patients (Galli et al., 2016). MANF may also well derive from unknown source in newly diagnosed T1D patients as MANF levels in sera of patients with established T1D are similar to the levels found in healthy subjects (Galli et al., 2016). Increased ER stress has been suggested to contribute to insulin resistance in adipose tissue, liver and muscle in obese, prediabetic and T2D mice and humans (Cnop et al., 2012). Thus, increased MANF levels in sera from prediabetic and T2D patients may derive from these tissues or endothelial cells in the blood vessels.

## MANF Expression Is Differently Regulated in Beta Cells Upon Stress-Induction

Cytokines were found to upregulate MANF expression in human islet beta cells and EndoC-βH1 cells *in vitro* (Hakonen et al., 2018). In contrast, decreased MANF expression and increased MANF protein degradation was seen in rat insulinoma INS-1E cell line treated with cytokines or chemical ER stressors (Cunha et al., 2017). Thus, there might be a species difference or genetic impact in the level of MANF expression by different cell stressors. In immune-independent NOD mice, decreased expression of *Glis3* was found to be responsible for the reduced *Manf* expression which led to beta cell death caused by increased ER stress due to overexpression of the hen egg lysozyme (Dooley et al., 2016). Thus GLIS3 transcription factor is implicated to regulate MANF expression in beta cells. GWAS have identified *GLIS3* as a known risk gene for T1D and T2D (Calderari et al., 2018). Patients with mutations in this gene show neonatal diabetes, skeletal and other defects. Importantly, *Glis3^-/-^* mice become severe diabetic neonatally due to reduced beta cell mass (Watanabe et al., 2009). In a genome-wide chromatin immunoprecipitation sequencing screen aimed at searching for Glis3 binding sites in INS-1 cells, 199 putative genes were found to bind Glis3 (Calderari et al., 2018). Interestingly, genes with Glis3 binding sites were enriched for GWAS loci associated with metabolic diseases and neuropathologies. Consequently, GLIS3 seem to regulate genes involved in the function of the endocrine pancreas and neurons. Although, reduced *Manf* expression was followed by defect Glis3 expression in diabetes susceptible NOD mice (Dooley et al., 2016), the *Manf* gene was not found in the list of Glis3 binding genes (Calderari et al., 2018). This suggests that GLIS3 is not directly responsible for the regulation of MANF expression or a functional Glis3 binding site is not present in the rat *Manf* gene.

## Protective Effect of MANF in ER Stress-Induced Apoptosis

Extensive beta cell apoptosis was detected in *Manf^-/-^* beta cells at 2 weeks of age (**Table 1**, Lindahl et al., 2014), probably due to chronic or T-UPR in *Manf^-/-^* beta cells leading to beta cell death. In contrast, MANF-overexpression in beta cells of diabetic mice partly protected against streptozotocin-induced beta cell death even though virus-delivered MANF transduction efficacy was low (∼4% of beta cells) (**Table 1**, Lindahl et al., 2014). Streptozotocin is a cytotoxic glucose analog that enters beta cells through GLUT2 and selectively kills beta cells and induces insulitis in rodents commonly used to mimicking T1D in humans (Lenzen, 2008). Importantly, recombinant human (rh)MANF partly rescued isolated mouse beta cells from thapsigargin- and cytokine-induced beta cell death *in vitro* (**Table 1**, Cunha et al., 2017). In contrast, MANF failed to protect clonal rat INS-1E cells from cytokine- and thapsigargin-induced apoptosis (**Table 1**, Cunha et al., 2017). In fact, *Manf* mRNA depletion from INS-1 cells by siRNA inhibition aggravated beta cell death induced by stressors (**Table 1**, Cunha et al., 2017). In this study MANF mediated cytoprotection of beta cells through thrombospondin 1. THBS are Ca^2+^-binding glycoprotein induced at sites of injury. Recently, THBS proteins showed ER stress protecting properties in cardiomyocytes and beta cells exposed saturated fatty acids (Lynch et al., 2012; Cunha et al., 2016). Overexpression of THBS1 in mouse and human beta cells and rat INS-1 cells partially protected against thapsigargin- and cytokine-induced intrinsic beta cell apoptosis whereas islets from *Thbs^-/-^* mice showed increased vulnerability against the same stressors (Cunha et al., 2017). ER stress and cytokines were shown to downregulate THBS1 expression in human and mouse beta cells and in INS-1 cells. This was suggested to depend on reduced MANF expression and thus MANF mediating the protective effect of THBS1 in these cells. As BH3-only proteins are important in mediating cytokine-induced beta cell apoptosis, BIM but not DP5, PUMA or BAD, seemed to be the downstream mediator of beta cell death in cytokine- and thapsigargin- treated THBS1 and MANF siRNA silenced INS-1 cells (Cunha et al., 2017).

**Table 1:** Survival and proliferation of beta cells.

Cell type	Survival						Proliferation	
		
	No factor		Thapsigargin		Cytokines		No factor	MANF
				
	No factor	MANF	No factor	MANF	No factor	MANF		
Human beta cells	-	-	n/a	n/a	↓^1,2^	↑^1,2^	-	↑ TGF-β + inhibitor^1^
EndoC-βH1 cells	-	-	↓^2^	↑^2^	↓^1,2^	↑^1,2^	n/a	n/a
EndoC-βH3 cells	n/a	n/a	n/a	n/a	n/a	n/a	-	↑^1^
Human beta cells MANF siRNA	-	n/a	n/a	n/a	↓↓^2^	n/a	n/a	n/a
EndoC-βH1 cells MANF siRNA	-	n/a	↓↓^2^	n/a	↓↓^2^	n/a	n/a	n/a
Mouse beta cells (young mice)	-	-	↓^2^	↑^2^	↓^2^	↑^2^	-	↑^3^
Mouse Manf KO beta cells^∗^	↓^3^	n/a	n/a	n/a	n/a	n/a	↓^3^	n/a
AAV-MANF islet beta cells^∗∗^	STZ	STZ	n/a	n/a	n/a	n/a	STZ	STZ
	↑^3^	↓^3^					↓^3^	↑^3^
INS-1E cells	-	n/a	↓^2^	↓^2^	↓^2^	↓^2^	n/a	n/a
INS-1E cells MANF siRNA	-	n/a	↓↓^2^	n/a	↓↓^2^	n/a	n/a	n/a


*↑, survival or proliferation increased; ↓, survival or proliferation decreased; ↓↓, aggravated death; -, no effect; n/a, data not available; ^∗^, *in vivo;*^∗∗^*, in vivo* in streptozotocin-induced T1D mouse pancreas. ^*1*^Hakonen et al., 2018; ^*2*^Cunha et al., 2017; ^*3*^Lindahl et al., 2014.*

Recombinant human MANF partly protected human EndoC-βH1 cells from both cytokine- and thapsigargin-induced cell death (**Table 1**, Cunha et al., 2017; Hakonen et al., 2018). These results were confirmed in EndoC-βH1 cells where rhMANF reduced the cytokine-induced cell death by 50% compared to control cells (Hakonen et al., 2018). Interestingly, rhMANF reduced endogenous *MANF* and *GRP78* mRNA expression in cytokine-treated EndoC-βH1 cells, suggesting that exogenously added MANF reduces ER stress and thereby downregulates the expression of ATF6 and XBP1s mRNA (Hakonen et al., 2018). These transcription factors are important inducers of *MANF* gene expression thus explaining the downregulation of endogenous MANF. MANF knockdown by siRNA in human primary beta cells and EndoC-βH1 cells intensified cytokine- and thapsigargin- induced beta cell death (**Table 1**, Cunha et al., 2017; Hakonen et al., 2018), suggesting that MANF is important for human beta cell survival. In addition to increased apoptosis in MANF-depleted cytokine-treated EndoC-βH1 cells, expression of UPR markers *ATF4, ATF3* and *CHOP* were increased (Hakonen et al., 2018). These results confirm the importance of exogenous and endogenous MANF for protecting mouse and human beta cells from ER stress-induced cell death. Rat INS-1 cells seemed to respond differently as MANF was not upregulated upon stress-induction and did not respond to exogenous human MANF (**Table 1**, Cunha et al., 2017), suggesting a species difference in MANF protection.

The mechanistic effect of MANF-induced protection of primary human beta cells to cytokine-induced cell death was studied by RNA sequencing of the transcriptome from islet preparations (Hakonen et al., 2018). Addition of MANF alone to human beta cells did not change gene expression significantly. The addition of a potent cytokine cocktail including IL-1β, IFN-γ, IL17 and TNF-α for 48 h, led to the upregulation of 618 genes and 377 downregulated genes compared to non-treated controls. Interestingly, addition of MANF together with cytokines resulted in an unexpected 15% increase in global gene expression or mRNA reads. As IRE1α is the first UPR pathway activated in *Manf^-/-^* mouse beta cells at E18.5 (Lindahl et al., 2014), exogenous MANF could thus suppress the IRE1α mediated RNA decay (RIDD) in cytokine-stressed islets leading to the increase in mRNA reads. However, further studies need to be performed to elucidate the mechanistic connection between MANF, IRE1α signaling and RIDD.

Addition of MANF to cytokine-treated human beta cells led to a significant upregulation of 251 genes whereas only one was downregulated. The only significantly downregulated gene was *BCL10*, which is a known inducer of apoptosis probably through NF-κB signaling inducing caspase 9 activation (Ruland et al., 2001; Mazzone et al., 2015). In contrast, knockdown of MANF by siRNA resulted in a significant increase in BCL10 expression and apoptosis in cytokine-treated EndoC-βH1 (Hakonen et al., 2018), thus implicating that MANF could regulate beta cell survival through regulation of BCL10 levels.

Differential expression analysis from the transcriptome data was performed after standard RNA-seq normalization and 282 differently expressed genes were found between the cytokines group compared to the MANF-treated cytokines group (Hakonen et al., 2018). Among these were genes related to cellular movement, proliferation and growth, gene expression and cell cycle. Interestingly, 30 from the 282 genes were found to be involved in NF-κB pathway. As activation of the NF-κB pathway in beta cells is associated with inflammatory response and cell death (Melloul, 2008), activation levels of NF-κB was studied in EndoC-βH1 cells. NF-κB is activated through IkappaB kinases (IKK)-mediated phosphorylation of RELA/p65 at Serine 536 and phosphorylation and degradation of inhibitory IκBα leading to nuclear translocation and transcriptional activation of NF-κB (Sakurai et al., 1999). MANF was found to partly inhibit the translocation of the RELA/p65 component to the nucleus in cytokine treated EndoC-βH1 cells (Hakonen et al., 2018). The decreased levels of phosphorylated Ser537 RELA/p65 in MANF-treated cells were confirmed by Western blotting. Importantly, these results indicated that MANF is able to reduce NF-κB inflammatory signaling and BCL10 induction in cytokine-stressed human beta cells.

MANF ability to regulate NF-κB signaling is supported by a recent study where MANF was shown to inhibit NF-κB induced transcriptional activation of inflammatory genes in fibroblast-like synoviocytes by binding to p65 and translocating into the nucleus, thus interfering with the binding activity of NF-κB to target promoters (Chen et al., 2015b). However, MANF has not been detected in the nucleus of stressed human or mouse beta cells tested by specific MANF antibodies confirmed on knockout tissue and cells (Hakonen et al., 2018). It has been shown that sustained PERK/pEIF2α activation leads to increased NF-κB translocation to the nucleus through decreased IκBα translation. In addition, increased IRE1α signaling leads to increased IKK phosphorylation and downstream p65 and IκBα phosphorylation and degradation (Zhang and Kaufman, 2008; Garg et al., 2012). Thus, previous results suggest that the mechanism behind MANF protection may lie in the inhibition of ER stress-induced reduction in NF-κB activation, BCL10 expression and apoptosis.

## Role for MANF in Pancreatic Beta Cell Proliferation

Under normal conditions the primary mechanism for maintaining the postnatal beta cells mass in adults is replication of existing beta cells (Dor et al., 2004), but basal beta cell proliferation and response to mitogenic triggers declines markedly with age in both rodents and humans (Teta et al., 2005; Kushner et al., 2010; Kushner, 2013). Interestingly, the beta cell mass is expanding during pregnancy in rodents and in obesity also in humans suggesting that beta cells can proliferate during adulthood. In fact, recent studies have identified factors that age-dependently regulate or limit beta cell proliferation in mice and human.

Expression of *EZH2*, a polycomb histone methyl transferase, declines with aging in beta cells (Zhou et al., 2013; Adamson et al., 2016). EZH2 has been found to repress cell cycle inhibitor *p16^INK4a^* and tumor suppressor *p19^ARF^* genes encoded by the *Cdkn2A* locus (Krishnamurthy et al., 2006; Chen et al., 2009). Thus decline in EZH2 and increase in expression of p16^INK4a^, a potent inhibitor of the proliferative kinase CDK4, leads to reduced beta cell proliferation with age (Krishnamurthy et al., 2006). A recent study using genetic mouse models revealed that platelet-derived growth factor receptor signaling (PDGFR) in beta cells induced *Ezh2* expression, delayed age-dependent Ezh2 loss and promoted beta cell expansion in adult mice (Chen et al., 2011). In addition, PDGFRa signaling resulted in ERK/CyclinD1 activation upregulating *EZH2* expression in human beta cells which led to increased proliferation through inhibition of p16^INK4a^. Thus, reduced PDGFR signaling may be one cause of age-dependent mitogenic restriction in beta cells. Other extrinsic signals that regulate beta cell proliferation include the lactogenic hormones PRL and PL. Both effectively stimulate proliferation of rodent beta cells *in vitro* and *in vivo* (Billestrup and Nielsen, 1991). However, no beta cell proliferation was detected in adult human islets with PRL and ectopic expression of PRL receptor in adult human beta cells did not restore responsiveness to PRL (Chen et al., 2015a). GLP-1 has an established role in stimulating insulin secretion and insulin biosynthesis in beta cells and rescue beta cells from apoptosis (Campbell and Drucker, 2013). The GLP-1 analog Exendin-4 (Ex-4) has been shown to induce mouse beta cell proliferation in an age-dependent manner in a streptozotocin-induced T1D mouse model (Tschen et al., 2009). The increased proliferation was associated with inhibition of the *p16^INK4a^* gene. Ex-4 was shown to induce proliferation in juvenile but not adult islets likely by failing to regulate the age-associated cell cycle inhibitor *CDKN2A* (Dai et al., 2017).

Embryonic deletion of MANF in pancreatic beta cells resulted in a significant abrubt neonatal decrease in beta cell proliferation whereas beta cell proliferation was not affected in *Manf^-/-^* mice before birth (**Table 1**, Lindahl et al., 2014). *In vivo*, overexpression of MANF in beta cells resulted in increased islet size in pancreases of diabetic mice compared to those that were transduced with control adeno associated virus 6 (AAV6)-RFP (**Table 1**, Lindahl et al., 2014). This was due to a significant increase in beta cell proliferation and decrease in beta cell death in the AAV6-MANF treated mice, demonstrating that overexpression of MANF could enhance beta cell proliferation and regeneration *in vivo*. However, we concluded that the low transduction efficiency (∼4%) was not enough to show a beneficial therapeutic effect restoring the blood glucose levels in mice overexpressing MANF in the beta cells. These results suggested that endogenous MANF is critically important for the proliferation of mouse beta cells.

The effect of exogenous rhMANF on primary mouse beta cells was assessed *in vitro*. Compared to islets cultured without added growth factors, rhMANF significantly increased proliferation of beta cells from young adult mice (**Table 1**, Lindahl et al., 2014).

Comparative analysis of the RNA sequencing data from the cytokine-treated primary human beta cells cultured in the presence or absence of MANF revealed clusters of upregulated genes in the MANF-treated cells with functions related to the regulation of G2/M transition of the mitotic cell cycle (Hakonen et al., 2018). Consequently, rhMANF proliferative effect was tested *in vitro* on adult human islets. Surprisingly, rhMANF together with a pharmacological TGF-β inhibitor SB431542 increased the proliferation of human beta cells by 2.5-fold shown by EdU incorporation compared to either MANF or TGF-β inhibitor alone (**Table 1**, Hakonen et al., 2018). Recently it was shown that TGF-β signaling via Smad3, activated or maintained *p16^Ink4a^* expression and thus led to replicative decline in adult mouse beta cells (Dhawan et al., 2016). In contrast, small molecule inhibition of TGF-β signaling reduced the expression of *p16^INK4a^* in human beta cells of islets grafted under the kidney capsule of immune deficient mice, thus resulting in increased proliferation of human beta cells (Dhawan et al., 2016). Importantly, rhMANF seemed to promote human beta cell replication after relief of mitogenic repression and rejuvenation of adult human beta cells by inhibition of TGF-β signaling (Hakonen et al., 2018). To further confirm the mitogenic effect of MANF on human beta cells, a 3rd generation human EndoC beta cell-line, EndoC-βH3 that phenotypically is very close to genuine adult human beta cells, was used (Benazra et al., 2015). Interestingly, MANF addition to the quiescent EndoC-βH3 cells alone resulted in a 3-fold increase in proliferation, which was not potentiated by SB431542 (**Table 1**).

Thus, in contrast to many mitogens effective for mouse beta cells, but not human beta cells, MANF was shown to have a mitogenic effect on human beta cells which could be potentiated by inhibition of TGF-β signaling pathways. In addition, extracellular MANF had a direct proliferative effect on mouse primary beta cells and quiescent human EndoC-βH3 cells capable of mediating intracellular mitogenic signaling. Importantly, the beta cell is the only cell type so far shown to respond to MANF by mitogenic action in culture.

## MANF Hypothetical Mode of Action

It is evident that endogenous MANF is needed for the survival and proliferation of mouse beta cells as lack of MANF in beta cells results in ER stress and sustained UPR activation leading to reduced expression of beta cell specific genes, decreased beta cells proliferation and increased cell death (Lindahl et al., 2014). The mechanism whereby endogenous MANF acts is not known. MANF expression and secretion is increased upon increased ER stress in mouse and human beta cells. This implies that it acts in two ways (1) by directly regulating the intensity and duration of ER stress trying to restore the ER homeostasis, or if not successful to alleviate ER stress, (2) MANF is secreted to act as a autocrine/paracrine factor trying to rescue the particular cell itself and nearby cells. It could re-enter cells or neighboring cells, translocate back to the ER, thus relieving ER stress, avoiding T-UPR and apoptosis. Exogenous MANF could also induce intracellular signaling cascades in the cell by binding and activating cell membrane receptor(s) leading to activation of unidentified signaling cascades for beta cell survival and proliferation (Stewart et al., 2015).

As exogenous MANF was found to reduce ER stress and was both mitogenic and protective for human and mouse beta cells in culture (Lindahl et al., 2014; Hakonen et al., 2018), it is likely that MANF is able to act in an autocrine/paracrine manner and somehow enter beta cells. However, the question remains how MANF exerts its beneficial effects. The identification of signaling receptors for MANF has been challenging. MANF was found to bind weakly to the KDEL receptor in the ER with its C-terminal amino acid consensus sequence RTDL that resembles the canonical ER retention signal, KDEL (Henderson et al., 2013). The same study suggested that MANF also binds KDEL receptors at the cell membrane of cell-lines overexpressing KDELRs (Henderson et al., 2013). However, direct binding of MANF to KDELR has not been shown. Recently, MANF was shown to bind to lipid sulfatides (e.g., 3-*O*-sulfogalactosylceramide) located at the outer leaflet of the membrane of *C. elegans* and in mammalian cells following MANF uptake by endocytosis into the cells (Bai et al., 2018). Thus, sulfatides have been proven important for MANF cell surface binding, transport and secretion (Bai et al., 2018). Sulfatides are synthesized in beta cells but not in pancreatic exocrine acinar cells (Boslem et al., 2012) and the N-terminal domain of MANF contains a saposin-like domain known to be able to bind lipids (Parkash et al., 2009), therefore exogenously added MANF might be taken up by beta cells through lipid sulfatide binding and transported back to ER where it relieves ER stress leading to increased proliferation and/or protection from ER stress-induced apoptosis. However, the relatively robust mitogenic effect of MANF on beta cells might still depend on yet unidentified signaling receptor(s) and signaling cascades.

**FIGURE 3:**
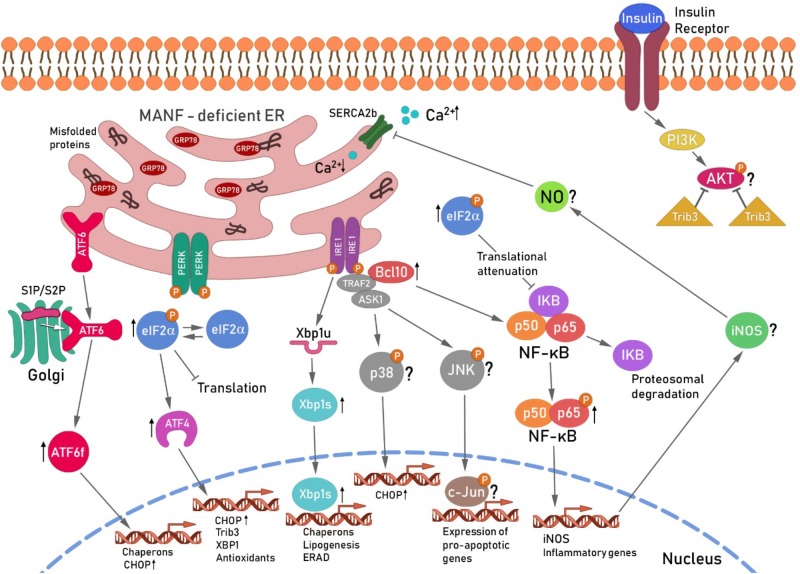
Hypothetical scheme for the unfolded protein response-induced signaling cascade in MANF-deficient beta cells. Ablation of MANF in mouse beta cells *in vivo* leads to sustained UPR, increased inflammatory signaling and beta cell death. The IRE1α pathway, *Xbp1s* and *Chop* mRNA levels were found increased in the pancreases of *Manf^-/-^* embryos at E18.5, whereas the PERK and ATF6 pathways were upregulated and chronically activated after birth in *Manf^-/-^* islets (Lindahl et al., 2014). Chronic activation of IRE1α and its oligomerization, has been shown to recruit TRAF2 and ASK1 in a complex that activates JNK and p38 signaling cascades, leading to increased expression of CHOP and pro-apoptotic genes (Ron and Hubbard, 2008; Brozzi and Eizirik, 2016). IRE1α/TRAF2 complex also stimulates the activation of pro-inflammatory NF-κB signaling cascades via IκBα proteosomal degradation by ERAD leading to intrinsic beta cell death. BCL10 expression levels were found decreased in cytokine-treated human beta cells and silencing of MANF in cytokine-treated human EndoC-βH1 resulted in a significant increase of BCL10 expression (Hakonen et al., 2018), suggesting involvement of BCL10 in the beta cell survival pathway regulated by MANF. BCL10 is a known inducer of apoptosis and an upstream regulator of NF-κB and also a binding partner of TRAF2 (Yoneda et al., 2000). Thus, BCL10 was placed with TRAF2 activated by IRE1α in the scheme. NF-κB nuclear translocation induces among other genes, iNOS expression leading to subsequent NO formation. NO constrain the SERCA2b pump, consequently leading to depletion of Ca^2+^ from the ER, resulting in increased ER stress (Fonseca et al., 2011) and cell death. The role of the JNK/p38 pathway in *Manf^-/-^* islets is under investigation. Activation of ATF6 pathway results in enhanced expression of pro-apoptotic transcription factor CHOP. The activation of PERK pathway followed by phosphorylation of eIF2α leads to global inhibition of protein synthesis and selective increase in translation of transcription factor ATF4. EIF2α was chronically phosphorylated and ATF4 mRNA upregulated in islets from *Manf^-/-^* mice (Lindahl et al., 2014). Global inhibition of translation induced by pEIFα also leads to reduced expression of inhibitory IκBα resulting in increased NF-κB nuclear translocation and activation of inflammatory genes. ATF4 is known to induce the expression of *Chop, Trib3* and *Xbp1*. Trib3 has been shown to inhibit AKT phosphorylation in the insulin signaling pathway (Du et al., 2003). In addition, *Trib3* expression was shown to be reduced in beta cells with genetically reduced insulin production due to reduced PERK/eIF2α/ATF4 activation (Szabat et al., 2016). Reduced ATF4 led to increased AKT Ser473 phosphorylation and increase in beta cell proliferation via cyclinD1. Thus decreased beta cell proliferation in *Manf^-/-^* mice could be caused by increased ATF4-induced *Trib3* mRNA expression and increased inhibition of AKT Ser473 phosphorylation and insulin signaling. ER, endoplasmic reticulum; ATF6, activating transcription factor 6; S1P and S2P, site 1 and 2 proteases; ATF6f, cytosolic fragment of ATF6; PERK, protein kinase RNA(PKR)-like endoplasmic reticulum kinase; eIF2α, eukaryotic translation initiation factor 2α; Trib3, pseudokinase tribbles homolog 3; PI3K, phosphatidylinositide 3-kinases; AKT, protein kinase B; IRE1, inositol-requiring protein 1; TRAF2, TNF receptor associated factor 2; ASK1, apoptosis signal-regulating kinase-1;NF-κB, nuclear factor κ-light-chain-enhancer of activated B cells; JNK, c-Jun N-terminal kinases, BCL10, B-cell lymphoma/leukemia 10; IκB, inhibitor of κB kinases; iNOS, inducible nitric oxide synthase: NO, nitric oxide; SERCA2b, sarcoplasmic/endoplasmic reticulum calcium ATPase. This scheme is modified from Danilova et al. (2018).

MANF-depletion from mouse and human beta cells leads to chronic ER stress and sustained upregulation of all UPR branches (Lindahl et al., 2014) (**Figure 3**). Sustained IRE1α activates TRAF2 and thereby contributes to NF-κB and JNK activation (Urano et al., 2000; Brozzi and Eizirik, 2016). BCL10 is a known inducer of apoptosis and upstream regulator of NF-κB signaling but also a binding partner of TRAF2 (Yoneda et al., 2000; Ruland et al., 2001; Mazzone et al., 2015). As NF-κB activation was found to be relieved and BCL10 mRNA expression reduced by MANF addition in cytokine-treated human beta cells (Hakonen et al., 2018), one could speculate that the protective mechanism of exogenously added MANF is to reduce ER stress and thereby block the inflammatory signaling pathways NF-κB and JNK that leads to beta cell death. MANF has been linked to inflammation also in other cell types. MANF inhibited NF-κB induced transcriptional activation of inflammatory genes in synoviocytes (Chen et al., 2015b). In addition, MANF was found to inhibit oxygen-glucose deprivation-induced cell damage and inflammatory cytokine secretion by suppressing ER stress in rat primary astrocytes (Zhao et al., 2013). Interestingly, PDGF AA released from injured retina promoted MANF expression in innate immune cells, and biased cells toward anti-inflammatory phenotype thereby promoting retinal tissue repair (Neves et al., 2016). Thus, results in beta cells so far support a mechanistic link between MANF, ER stress and inflammatory pathways. Importantly, as MANF has been found to regulate insulin signaling in the hypothalamus, it might as well regulate insulin receptor signaling through PERK/eIF2α/ATF4/Trib3 modulation of AKT phosphorylation in the beta cells. We propose that MANF-deficiency or downregulation of MANF in beta cells might hasten beta cell demise by chronic ER stress leading to increased inflammatory signaling and reduced insulin expression and insulin signaling (**Figure 3**). Exogenous MANF thus might alleviate ER stress, inflammatory signaling and increase insulin signaling and AKT phosphorylation leading to increased beta cell proliferation. However, evidence for this hypothesis is lacking and MANF mechanism of action in beta cells needs further investigation.

## Conclusion and Prospects for MANF as Regenerative Therapeutic Agent in Diabetes

Therapeutic strategies directed to induce endogenous beta cell regeneration are currently under intensive investigation. A number of strategies have proven effective in inducing regeneration of mouse and human beta cells. These involve initiation of beta cell proliferation and transdifferentiation of different pancreatic cells to functional beta cells (Aguayo-Mazzucato and Bonner-Weir, 2018). Despite great advances in understanding mechanisms underlying regeneration in different approaches and identifying small drugs to enhance regeneration, there are limitations and concerns in their use.

Targeting ER stress and the UPR in diabetes is a promising approach for beta cell regeneration. A number of pharmacological compounds and small molecules are available to modulate ER stress and the UPR. Low molecular weight chemical chaperons 4-phenyl butyric acid (PBA) and TUDCA were shown to reduce phosphorylation of PERK and IRE1α and downstream JNK in cells from ob/ob mice and normalized hyperglycemia and insulin resistance in those mice (Ozcan et al., 2006). In addition, TUDCA reduced the incidence of diabetes and insulitis in NOD mice as well as improved insulin secretion and beta cell morphology (Engin et al., 2013). In humans, oral PBA treatment for 2 weeks partially ameliorated intralipid infusion-induced beta cell dysfunction thus suggesting that PBA might reduce insulin resistance in T2D patients (Xiao et al., 2011). Recent inventions have led to the discovery of several new small molecules that target the enzymatic activities of UPR sensors. IRE1 inhibitors that selectively inhibit IRE1’s RNase activity were found to reduce ER stress-induced inflammation and atherosclerosis in hyperlipidemic mice (Tufanli et al., 2017). Several studies have shown that Ex-4 used to treat T2D, is protective for beta cells via modulating ER stress induced by tunicamycin, thapsigargin, saturated fatty acids and glucolipotoxic conditions (Yusta et al., 2006; Cunha et al., 2009; Oh et al., 2013). *In vivo*, Ex-4 treatment of diabetic *Ins2^Akita^* mice showed attenuated ER stress, associated with reduced beta cell death and lowered blood glucose levels (Yamane et al., 2011). Thus, these studies and others hold promise for GLP-1 receptor agonists in the treatment of T1D patients.

Cytosolic ABL kinases were found to interact with IRE1α in the ER and to induce hyperactive IRE1α RNase activity (Morita et al., 2017). This interaction was reduced by the anti-cancer drug imatinib, *in vitro* and diabetes was prevented and reversed in NOD mice. The anti-diabetic effect of imatinib was suggested to rely on blunting the T-UPR through decreased IRE1α/ABL interaction, reduced IRE1α-dependent RNase activity and downregulation of TXNIP levels. In addition mono-selective IRE1 kinase/RNase inhibitors, KIRAs were able to nearly completely reverse established diabetes in the NOD and Ins2^Akita^ mouse models. The recovery was accompanied with reduced TXNIP expression and increased *Manf, Grp78* and *Ins1/Ins2* mRNA expression. Thus, T-UPR plays a central role in degeneration of beta cells in T1D and temporal revival of beta cell function after starting insulin therapy may be caused by reduced beta cell ER stress and T-UPR. Thus researchers speculate that there are salvageable beta cells during this “honeymoon period” that could be a promising target for the treatment of T1D with drugs that blunt the ABL-IRE1α or other UPR branches (Morita et al., 2017). As MANF was found upregulated in T1D beta cells under A-UPR and down-regulated at the time of initiation of T-UPR onset, increasing MANF levels in beta cells could hinder beta cell transition from A-UPR to T-UPR and cell death executive pathways. Thus MANF could be a potential therapeutic factor to alleviate ER stress, rescue beta cells and induce beta cell regeneration in diabetes.

The data discussed in this review suggest that MANF is a novel potent beta cell protective and mitogenic factor for human and mice beta cells. The mechanism of action for MANF is still elusive but point to a regenerative effect acting both intracellularly and exogenously through modulating sustained ER stress in beta cells, which is an important factor contributing to beta cell dysfunction and death leading to insulin-deficiency in T1D and T2D. Thus MANF alone or in combination with other drugs is a potential agent for development into a regenerative drug for beta cells in diabetes. From the translational and clinical point of view, results with mice suggest that MANF could be delivered to diabetic human pancreas and beta cells through viral AAV vectors by endoscopic non-surgical procedures. However, clinical trials using AVV vectors will uncover whether the use of these vectors are efficient and safe. Future studies will reveal whether MANF protein-based systemic delivery can normalize blood glucose levels in rodent models of diabetes. In addition, efforts to improve MANF stability and efficacy will be required for translating the MANF therapy to humans.

## Ethics statement

All experimental procedures involving mice were approved by the Finnish Animal Ethics Committee.

## Author Contributions

TD wrote the manuscript partly and constructed the figures. ML wrote and edited the manuscript, constructed and planned the figures.

## Conflict of Interest Statement

The authors declare that the research was conducted in the absence of any commercial or financial relationships that could be construed as a potential conflict of interest. The reviewer DL declared a shared affiliation, with no collaboration, with the authors to the handling Editor at time of review.

## Abbreviations

AAV: Adeno-associated virus
APAF1: apoptotic protease activating factor-1
ATF4: activating transcription factor 4
A-UPR: adaptive unfolded protein response
BAK: Bcl-2 homologous antagonist/killer
BAX: Bcl-2-associated X protein
BCL2: B cell lymphoma
CDK4: cyclin dependent kinase 4
CDNF: cerebral dopamine neurotrophic factor
CHOP: C/EBP homologous protein-10
CRISPR: clustered regularly interspaced short palindromic repeats
DM: diabetes mellitus
E18.5: embryonic day 18.5
ER: endoplasmic reticulum
ERAD: ER-associated protein degradation
Ex-4: exendin 4
EZH2: zeste homolog 2
FFA: free fatty acid
GDNF: Glial cell line-derived neurotrophic factor
GLIS3: Gli-similar 3 protein
GLP-1: glucagon-like peptide-1
Glut2: glucose transporter 2
GRP78: 78-kDa glucose-regulated protein
GWAS: genome-wide association studies
IAPP: islet amyloid polypeptide
IFNγ: interferon γ
IL1β: interleukin 1β
MafA: musculoaponeurotic fibrosarcoma oncogene family A
MANF: Mesencephalic astrocyte-derived neurotrophic factor
p38 MAPK: p38 mitogen-activated protein kinases
NOD: non-obese diabetic mice
NRF2: nuclear factor erythroid 2-related factor 2
PARP: poly(ADP-ribose)polymerase
Pax6: paired box protein 6
PBA: 4-phenyl butyric acid
PDGFR: platelet-derived growth factor receptor
Pdx1: pancreas/duodenum homeobox protein 1
PIP4k2b: phosphatidylinositol 5-phosphate 4-kinase type-2 beta
PL: placental lactogen
P1: postnatal day 1
PP1: protein phosphatase 1
PRL: prolactin
RFP: red fluorescent protein
rhMANF: recombinant human MANF
RELA: v-rel avian reticuloendotheliosis viral oncogene homolog
RIDD: IRE1-dependent RNA decay
ROS: reactive oxygen species
STZ: streptozotocin
TGF-β: transforming growth factor β
THBS: thrombospondin proteins
TNF-α: tumor necrosis factor α
TXNIP: thioredoxin-interacting protein
TUDCA: taurine-conjugated ursodeoxycholic acid
T-UPR: terminal unfolded protein response
T1D: Type 1 diabetes
T2D: Type 2 diabetes
UPR: unfolded protein response
WFS1: Wolfram syndrome 1
WT: wild type
